# A rare variant of double origin of the left vertebral artery

**DOI:** 10.1007/s12565-025-00836-3

**Published:** 2025-04-12

**Authors:** George Triantafyllou, Panagiotis Papadopoulos-Manolarakis, Fabrice Duparc, George Tsakotos, Maria Piagkou

**Affiliations:** 1 Department of Anatomy, School of Medicine, Faculty of Health Sciences, National and Kapodistrian University of Athens, 75 Mikras Asias Str., Goudi, 11527 Athens, Greece; 2 Department of Neurosurgery, General Hospital of Nikaia-Piraeus, Athens, Greece; 3 Department of Anatomy, Faculty of Medicine-Pharmacy, University of Rouen-Normandy, Rouen, France

**Keywords:** Vertebral artery, Double origin, Aortic arch, Anatomy, Variation

## Abstract

The vertebral artery (VA) demonstrates considerable variability, including infrequent morphologic variants like double origin. The current imaging report presents an atypical case of a dual origin of a left vertebral artery (LVA), which was identified incidentally during routine computed tomography angiography of a 51-year-old male patient. The typical LVA originated from the left subclavian artery (LSCA), and the other one was an aberrant LVA (ALVA) that arose from the aortic arch (AA) in between the left common carotid artery (LCCA) and the LSCA. The two vessels (LVA and ALVA) converged proximally to the transverse process of the C6 vertebra. The embryological explanation for this variant likely pertains to the persistence of primitive dorsal aortic segments and intersegmental arteries. Although rare, with a prevalence of 0.1%, the occurrence of LVA double origin may have significant clinical implications, including alterations in hemodynamics, an increased risk of VA dissection, and complications during surgical or endovascular procedures. The identification of such variants through preoperative imaging is essential to prevent iatrogenic complications.

## Introduction

The ascending aorta continues as the left-sided AA (LAA), which gives rise to the brachiocephalic trunk (BCT), the left common carotid artery (LCCA), and the left subclavian artery (LSCA), reflecting the typical branching pattern observed in approximately 80.9% of the population (Popieluszko et al. 2018; Natsis et al. 2021). Notably, this typical branching pattern corresponds to the LAA, as a right-sided AA can also occur extremely rarely (pooled prevalence of 0.01%). (Triantafyllou et al. 2024). The CCA bifurcates into the external and internal carotid arteries. The internal carotid artery and vertebral artery (VA) supply blood to the brain. VA originates bilaterally from the SCA. Shortly after their origin, the VAs course into the transverse foramen (TF) of the sixth cervical vertebra (C6), most commonly (Lazaridis et al. 2018; Tudose et al. 2023).

The AA and VA morphologic variability is exceptionally high, with frequent and rare variants described. Tudose et al. (2023) calculated the pooled prevalence of the left VA (LVA) origin from the LSCA in 94.1% and the LAA in 4.81%. Contrariwise, the right VA (RVA) origin is significantly more constant from the LAA in 99.9% of the population (Tudose et al. 2023). Rarer variations are also recorded, such as the VA duplicate (double or dual) origin (Polguj et al. 2013b).

Here, we present a rare LVA double-origin variant identified incidentally during routine computed tomography angiography (CTA). We discuss the clinical significance and intriguing embryological development further.

## Anatomic variation

The exceptional arterial variant was identified during a retrospective CTA study of a Greek adult population sample (affiliation 2). Due to its unique morphology, the carotid CTA of a 51-year-old male patient was further examined. The investigation was conducted and documented using Horos software (Horos Project). Evidence was obtained from the multiplanar reconstruction of the axial, coronal, and sagittal slices and their three-dimensional volume reconstruction.

**Fig. 1:**
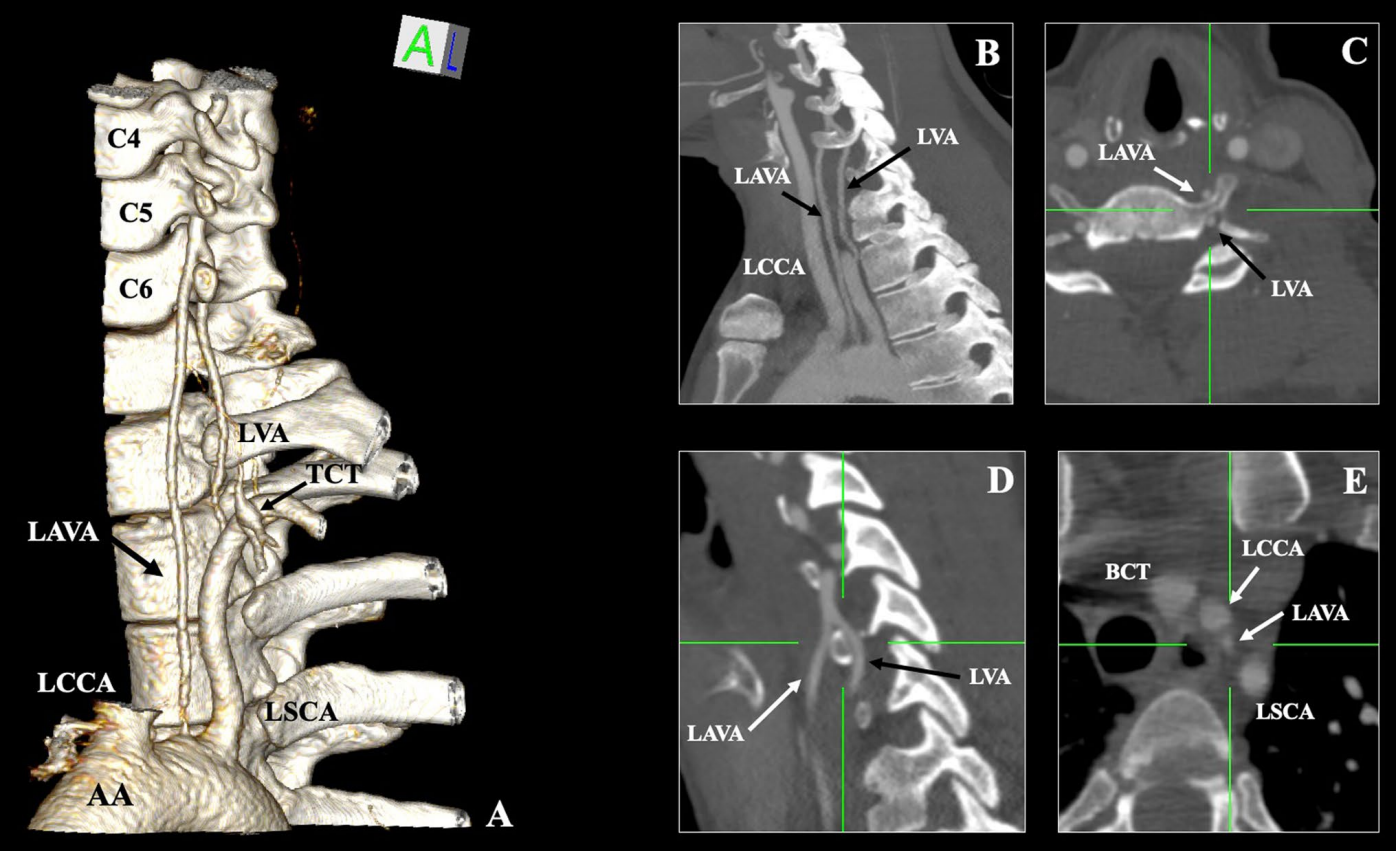
**A** Three-dimensional reconstruction depicting the dual origin of left vertebral artery (LVA), the one typically emanating from the left subclavian artery (LSCA) and the other- the aberrant vertebral artery (LΑVA) emanating from the left-sided AA on between the origins of the LCCA (left common carotid artery) and the LSCA. **B** Sagittal section with thick slices technique (maximum intensity projection) depicting the LVA and LAVA; **C** axial section at the level of sixth cervical vertebra (C6) depicting the LAVA anterior to the C6 and the LVA into the transverse foramen; **D** Sagittal section at the level of C6 depicting the fusion of the LVA and LAVA; **E** axial section at the aortic arch branches origin with the brachiocephalic trunk (BCT)

Four branches emerged from the LAA: the BCT, the LCCA, a variant branch, and the LSCA. When tracing the ascending course of the variant (aberrant) branch, it was positioned anterior to the transverse process (TP) of the thoracic and lower cervical vertebrae. At the C6 TP level, the variant (aberrant LVA-ALVA) branch fused with the LVA (typically originated from the LSCA) after its course through the C6 TF. Therefore, the atypical vessel corresponded to an LVA double origin (one from the LAA and another from the LSCA) (Fig. 1).

The typical LVA exhibited a diameter of 2 mm at its origin and a length of 48.6 mm until its entrance into the TF. In contrast, the ALVA exhibiting an (aberrant or ectopic) origin from the LAA presented a diameter of 1.9 mm at its origin and extended to a length of 96.4 mm before fusing with the typical LVA above the C6 TF. Following the confluence of the two origins, the LVA’s diameter increased to 2.9 mm.

The right side of the patient exhibited a typical VA without an aberrant branch.

## Discussion

In the current imaging report, LVA double origin was noted during the CTA scan of a 51-year-old male patient. Previous studies suggest that this variant can be easily justified by the AA embryological process (Padget 1948; Uchino et al. 2013). This procedure is intricate, particularly for the VA, as it transitions from a segmental type to a single vessel configuration. Six embryonic pairs of AAs are initially present, which will ultimately regress, resulting in only the residual branches forming the adult aorta. Modifying this complex procedure may result in morphologic variants (Lazaridis et al. 2018). The VA emanates from the distal end of the 7th dorsal intersegmental artery (DISA), leading to the proximal end of the vessel when the limb bud is developed (Padget 1948). Most commonly, the persistence of aberrant anastomosis leads to variants in VA origin, such as the AA origin that results from the persistence of the 6th DISA (Padget 1948). Prevertebral duplication, as seen in this case, may arise from the persistence of the primitive dorsal aorta with two intersegmental vessels connected to the VA or from the incomplete regression of the 5th or 6th intersegmental artery, which adds origin to the typical VA of the 7th intersegmental segment (Padget 1948; Polguj et al. 2013b). Therefore, these two theories lead to the formation of the VA double origin. Due to the entrance of the aberrant vessel from the C5 TF, we can hypothesize that this case corresponds to the incomplete regression of the 5th intersegmental artery (Jung et al. 2016).

*Bergman’s Comprehensive Encyclopedia of Human Anatomic Variation* describes the VA double origin as a rare variant that occurs unilaterally with equal frequencies for both LVA and RVA. Both vessels can originate from the SCA, one from the SCA while the other from the AA or the BCT. Most often, the two vessels fuse at the level between C5 and C6 (Tubbs et al. 2016). In the current literature, three VA variants are frequently confused: the “accessory VA,” the “VA double origin,” and the "VA fenestration” must be distinguished (Polguj et al. 2013b; Bordes et al. 2021). The “VA double origin,” which is also mistakenly defined as “VA duplication,” corresponds to the VA that has two origins that eventually fuse at different levels of the neck (Polguj et al. 2013b; Bordes et al. 2021). In a large retrospective study involving 2287 patients, Uchino et al. (2013) documented two cases of LVA double origin (0.08% prevalence) and one case of RVA double origin (0.04% prevalence). In both cases described by Uchino et al. (2013), the typical LVA arose from the LSCA, and the ALVA arose from the LAA, similar to the current case (Uchino et al. 2013). Lazaridis et al. (2018) investigated variants of VA origins during their thorough literature review. A total of 14,738 patients recorded 587 cases of atypical LVA origin (4%). They identified 16 cases of double origin of LVA, reporting a prevalence of 0.1%, indicating a rare variant (Lazaridis et al. 2018). Thirteen (13) cases had a dual origin from the LSCA and the LAA, two (2) from the LSCA and the thyrocervical trunk, and one (1) from the LSCA and the left external carotid artery. This last case could also be attributed to carotid-vertebrobasilar anastomosis, a different type of variant (Lazaridis et al. 2018). However, nine (9) cases were recorded with cadaveric dissection, and seven (7) cases using CTA (Lazaridis et al. 2018). The RVA double origin was even rarer, with four (4) cases described in the review by Lazaridis et al. (2018). Furthermore, a case reported by Watanabe et al. (2016) described a dual origin of the LVA, with one branch arising from the LAA and another from the LSCA, entering different cervical TF without convergence (Watanabe et al. 2016).

When VA duplication occurs in any form, whether partially (double origin) or completely, morphologic changes in the arterial wall can lead to factors contributing to vertebral dissection. Clinical presentations of posterior circulation insufficiency include vertigo, dizziness, or occipital heaviness, which can arise from any form of VA duplication due to alterations in the lumen (Polguj et al. 2013b). Changes in hemodynamics caused by arterial wall deformations can result in fenestration, kinking, and aneurysm formation (Suzuki et al. 1978). Nevertheless, Polguj et al. (2013b) described a dual origin of the LVA coexisting with dissection of the right internal carotid artery in a patient with Ehler–Danlos syndrome (Polguj et al. 2013a). Moreover, neurosurgeons performing anterior cervical approaches (such as diskectomies and corpectomies for trauma or degenerative disease), could potentially injure an aberrant VA during the lateral displacement/retraction of the longus colli muscles to expose the superficial disk space; therefore, preoperative imaging with CTA should always be utilized to evaluate the vascular anatomy, even for rarer variants (Polguj et al. 2013b; Yaman et al. 2024). The double-origin VA may influence the route choice during endovascular procedures, and the vessel with a larger luminal diameter may be preferable for the interventionist (Yaman et al. 2024).

## Conclusions

The presence of a double origin of the LVA is documented during a routine CTA scan of a 51-year-old male patient. The typical LVA originated from the LSCA, while the accessory origin arose from the LAA between the LCCA and the LSCA. The two vessels were fused above the TP of the C6 (distally to the C5-C6). Understanding arterial variations is essential prior to surgical and endovascular procedures to prevent iatrogenic injuries.

## Data Availability

Please get in touch with the authors for data requests (George Triantafyllou—email address: georgerose406@gmail.com).

## References

[CR1] Bordes SJ, Iwanaga J, Zarrintan S et al (2021) Accessory vertebral artery: an embryological review with translation from Adachi. Cureus. 10.7759/cureus.13448 33767932 10.7759/cureus.13448 PMC7982507

[CR2] Jung S, Jung C, Bae YJ et al (2016) Duplicated origin of the left vertebral artery: a case report and embryological review. Neurointervention 11:50. 10.5469/neuroint.2016.11.1.50 26958414 10.5469/neuroint.2016.11.1.50 PMC4781919

[CR3] Lazaridis N, Piagkou M, Loukas M et al (2018) A systematic classification of the vertebral artery variable origin: clinical and surgical implications. Surg Radiol Anat 40:779–797 29459992 10.1007/s00276-018-1987-3

[CR4] Natsis K, Piagkou M, Lazaridis N et al (2021) A systematic classification of the left-sided aortic arch variants based on cadaveric studies’ prevalence. Surg Radiol Anat 43:327–345. 10.1007/s00276-020-02625-1 33386933 10.1007/s00276-020-02625-1

[CR5] Padget DH (1948) Development of cranial arteries in human embryo. Contrib Embryol 32:205–262

[CR6] Polguj M, Jędrzejewski K, Topol M et al (2013a) Duplication of the left vertebral artery in a patient with dissection of the right internal carotid artery and Ehlers-Danlos syndrome: case report and review of the literature. Anat Sci Int 88:109–114. 10.1007/s12565-012-0152-z 22956231 10.1007/s12565-012-0152-z PMC3575557

[CR7] Polguj M, Podgórski M, Jędrzejewski K et al (2013b) Fenestration and duplication of the vertebral artery. Clin Anat 26:933–943. 10.1002/ca.22231 23553773 10.1002/ca.22231

[CR8] Popieluszko P, Henry BM, Sanna B et al (2018) A systematic review and meta-analysis of variations in branching patterns of the adult aortic arch. J Vasc Surg 68:298-306.e10. 10.1016/j.jvs.2017.06.097 28865978 10.1016/j.jvs.2017.06.097

[CR9] Suzuki S, Kuwabara Y, Hatano R, Iwai T (1978) Duplicate origin of left vertebral artery. Neuroradiology 15:27–29. 10.1007/BF00327442 643170 10.1007/BF00327442

[CR10] Triantafyllou G, Melissanidis S, Vlychou M et al (2024) Right-sided aortic arch: a computed tomography angiography investigation, a systematic review with meta-analysis. J Clin Med 13:3105. 10.3390/jcm13113105 38892815 10.3390/jcm13113105 PMC11172921

[CR13] Tubbs RS, Shoja MM, Loukas M (2016) Bergman’s comprehensive encyclopedia of human anatomic variation. Wiley, Hoboken

[CR14] Tudose RC, Rusu MC, Hostiuc S (2023) The vertebral artery: a systematic review and a meta-analysis of the current literature. Diagnostics 13:2036 37370931 10.3390/diagnostics13122036 PMC10296927

[CR15] Uchino A, Saito N, Takahashi M et al (2013) Variations in the origin of the vertebral artery and its level of entry into the transverse foramen diagnosed by CT angiography. Neuroradiology 55:585–594. 10.1007/s00234-013-1142-0 23344682 10.1007/s00234-013-1142-0

[CR16] Watanabe K, Saga T, Iwanaga J et al (2016) A rare case of dual origin of the left vertebral artery without convergence. Folia Morphol (Warsz) 75:136–142. 10.5603/FM.a2015.0064 26365864 10.5603/FM.a2015.0064

[CR17] Yaman V, Duzgun SA, Hazirolan T (2024) Dual origin of the left vertebral artery: a rare anatomic variation. Indian J Thorac Cardiovasc Surg 40:242–244. 10.1007/s12055-023-01641-1 38389782 10.1007/s12055-023-01641-1 PMC10879477

